# Unemployment & opioid use disorder – socio-medical assessment results by the Medical Service of the German Federal Employment Agency

**DOI:** 10.1186/s13011-026-00752-3

**Published:** 2026-07-29

**Authors:** Michael Soyka, Kirsi Manz, Andreas G. Franke

**Affiliations:** 1 Psychiatric Hospital, University of Munich, Nußbaumstr. 7, 80336 Munich, Germany; 2 Department of Hematology, Hemostasis, Oncology and Cell Therapy, Hannover Medical School (MHH), Hannover, Germany; 3 University of Applied Labour Studies, Federal Employment Agency, Mannheim, Germany

**Keywords:** Opioid use disorder, Opioid dependence, Comorbidity, Mental health disorders, Psychiatric disorder, Unemployment

## Abstract

**Background:**

The interrelationship between unemployment and substance use disorders, especially opioid use disorders, is complex, and basic epidemiological data are lacking both in Germany and elsewhere. This study aimed to evaluate the prevalence rates of opioid use disorder, work ability, and associated factors among unemployed individuals with opioid use disorder who underwent socio-medical assessment by the Medical Service (MS) of the Federal Employment Agency (FEA).

**Methods:**

All socio-medical assessments conducted between 2016 and 2021 (*N* = 4,249,028) were extracted from the FEA database and analyzed with respect to relevant sociodemographic characteristics, opioid use disorder (ICD-10 F11.x diagnosis) and work ability. Descriptive statistics were used to summarize the data.

**Results:**

A total of 4,249,028 unemployed individuals were assessed. Of these, 22,683 clients (0.5%) met an F11.x diagnosis (primary or secondary); mostly opioid dependence (*N* = 20,506; 94.3%). The number of diagnoses increased over time (2016–2021). Mean age was 40.7 ± 9.4 years. Overall, 15,065 (73.5%) were male and 5,441 (26.5%) were female. For individuals with a secondary diagnosis of F11.x, most frequent psychiatric comorbidities were alcohol dependence (27.8%), polysubstance use (17.6%), schizophrenia (8.8%), depression episode (8.3%) or recurrent depression (5.9%) and persistent pain disorder (6.1%). Regarding daily work capacity, 58.3% (*n* = 11,962) of individuals were able to work less than 3 h per day, 10.0% (*n* = 2,051) were able to work 3–6 h per day, and 17.4% (*n* = 3,568) were able to work more than 6 h per day.

**Conclusions:**

The number of unemployed individuals with opioid dependence in Germany has increased over time, and most are unable to work more than three hours per day. The most frequent psychiatric comorbidities were other substance use disorders, particularly alcohol use disorder, schizophrenia, and depression. Somatic comorbidities, with the exception of chronic viral hepatitis, were recorded less frequently. Tailored interventions and closer collaboration between employment agencies and mental health care providers are needed to improve the psychosocial integration of this population.

## Background

Opioid use is increasing worldwide [1, 2]. Recent data indicate a two-fold increase to 205/100,000 people [2]. In 2017, 40.5 million people worldwide were dependent on opioids [3]. Epidemiological studies indicate a global age-standardized prevalence of opioid use disorder (OUD) of about 0.5%. The number of opioid-related deaths worldwide was estimated at about 1.26 deaths per 100,000 people [4]. Prevalence estimates in the US suggest that some 4.8 million people use opioids [5].

While there are many studies on the somatic and neuropsychiatric sequelae of OUD and the associated burden of illness [1, 6], there are fewer data on the socioeconomic and social consequences of OUD [7–10].

Premature death due to overdose or other disorders is frequent in OUD [1, 6, 11–13], and many people suffer from multiple somatic and mental comorbidities [14]: OUD is associated with numerous somatic and neurological disorders such as hepatitis, HIV, endocarditis or other infections, brain damage (encephalopathy), injuries and other disorders. In addition, psychiatric disorders such as depression and anxiety as well as suicide are frequent [15–26]. There also is an excess risk for suicide [27]. Opioid agonist treatment (OAT) is the standard treatment in OUD with methadone and buprenorphine as first line medications with proven efficacy to suppress craving, opioid withdrawal symptoms and relapse in opioid users [28]. OAT is also effective at improving psychosocial functioning and health outcomes in OUD [1].

Mental disorders, including OUD, have a negative impact on work performance and increase workplace absences, thereby increasing the risk of unemployment and poor socioeconomic status over the work-life course [29–32]. On one hand, low psychosocial functioning and concurrent chronic somatic illness were identified as strong predictors of poor labour-market performance among people with severe mental disorders [33]. On the other hand, unemployment has been shown to have detrimental effects on mental health [34–36]. An extensive meta-analysis demonstrated that 34% of unemployed people suffer from mental health problems, a 2-fold higher prevalence compared to 16% among employed individuals [34].

In Germany, about 6.3% (*N* = 2,948,000) of the population is unemployed (January 2026) [37]. The Federal Employment Agency (FEA) is the principal authority for all labor market issues with an emphasis on job placement and labour-market integration for unemployed people (so called “clients”). Registered jobseekers are obliged to visit their employment agency regularly in order to document job search activities, get and accept job offers or undergo socio-medical assessments, if appropriate. Fundamentally, assessments are carried out on request of FEA counselors to determine an individual’s work ability and the extent to which clients can be integrated into the labor market. The evaluation is performed by medical practitioners from the Department of Medical Services (MS) of the FEA. The MS is responsible for about 500,000 socio-medical assessments per year with or without direct client interaction containing clinical examination and, in all cases, an in-depth medical history obtained by requesting medical records from practitioners (e.g., family doctors, specialists, hospitals, rehabilitation clinics) based on a patient’s confidentiality release [38, 39].

To date, systematic large-scale and current epidemiological data on the mental health status of unemployed individuals are scarce. A recent evaluation has shown general data about mental health [39, 40] but not regarding OUD. Basic data on OUD in Germany are still lacking, making it difficult to identify specific care needs and provide recommendations for the psychosocial management of affected individuals. Previous research has shown that unemployment is frequent in OUD and intravenous drug users [41–44].

The aim of this study was to systematically analyze all socio-medical assessment data from a predefined study period provided by the FEA and to evaluate the frequency of OUD, work ability, and associated socioeconomic data among unemployed people.

## Methods

Data basis and variables: This study is based on all socio-medical assessment data of the MS throughout Germany between 2016 and 2021 (*N* = 4,249,028), which represent the most recent data available for analysis. Data were extracted from the FEA database (COMEDSQL, Microsoft SQL Server 2016).

All clients of the FEA who underwent socio-medical assessment were asked to name all physicians and institutions that had provided treatment in the past and to release them from their duty of confidentiality. In compliance with data protection rules, treating physicians and institutions were asked to submit information about the respective patient/ client. Subsequently, experienced physicians with additional specialization in social medicine analyzed these data and, where appropriate, completed the assessment by performing a physical examination. The COMEDSQL database contains sociodemographic characteristics such as age (categorized into five-year intervals) and sex (male, female), diagnoses according to ICD-10 and an assessment of work ability according to three categories (< 3 h, 3 to 6 h, > 6 h per day). One main (primary) and one secondary diagnosis could be documented in the database; all diagnoses (including OUD) could be entered as primary and/ or secondary diagnosis. Primary diagnoses are considered the “leading” diagnosis having the main impact on all consequences (including ability/ performance to work). The diagnostic process to identify main diagnoses and comorbidities was carried out in accordance with the International Statistical Classification of Diseases and Related Health Problems, 10th revision (ICD-10) [45].

For this study, only data from clients with at least one diagnosis of the ICD-10 Chapter V: Mental and behavioral disorders with opioid use (F11.x) were included.

### Data analyses

Standard descriptive statistics were used to summarize the data. Continuous variables were summarized as means and standard deviations (SDs) and categorical variables were summarized as frequencies and percentages. For combined analyses (“main or secondary diagnosis”), each assessment record was counted once per diagnosis code, even if the same diagnosis was recorded in both positions. Temporal trends in diagnosis counts were assessed using Poisson regression models with calendar year entered as a continuous predictor. Regression coefficients (β), incidence rate ratios (IRRs) with their 95% confidence intervals (CIs), and p values derived from Wald tests were reported. All statistical analyses were performed using the software R, version 4.4.2.

### Data protection/ Ethics

In compliance with the guidelines of the Federal Commissioner for Data Protection and Information Security (BfDI), the study data did not contain any personally identifiable information. The study was approved by the Data Protection Department of the FEA (Ref. No. AZ1400.12-1/2023).

## Results

Between 2016 and 2021, a total of 4,249,028 socio-medical assessments were conducted. Of these, 22,683 assessments (0.5%) involving FEA clients with at least one opioid-related F.11.x diagnosis were identified and considered for further analysis. Overall, a mental disorder related to opioid use as first diagnosis was coded in 15,382 cases and as second diagnosis in 7,340 cases (see Table 1).

**Table 1:** Distribution of F11 diagnoses

Diagnosis	Main/ First diagnosis	Secondary diagnosis	Main or secondary
F11	93 (0.6%)	74 (1.0%)	167 (0.7%)
F11.0	71 (0.5%)	45 (0.6%)	116 (0.5%)
F11.1	610 (4.0%)	886 (12.1%)	1494 (6.6%)
F11.2	14,498 (94.3%)	6,045 (82.4%)	20,506 (90.4%)
F11.3	46 (0.3%)	178 (2.4%)	224 (1.0%)
F11.4	12 (0.1%)	2 (0.0%)	14 (0.1%)
F11.5	16 (0.1%)	67 (0.9%)	83 (0.4%)
F11.7	15 (0.1%)	20 (0.3%)	35 (0.2%)
F11.8	3 (0.0%)	1 (0.0%)	4 (0.0%)
F11.9	18 (0.1%)	22 (0.3%)	40 (0.2%)
**F11 total**	**15,382**	**7,340**	**22,683**

“F11” without subcode is retained as a separate category. Percentages refer to column totals. Codes following the ICD-10

Opioid dependence (F11.2) accounted for the vast majority of F.11.x diagnoses (acute intoxication, harmful use, withdrawal state with/ without delirium, psychotic disorder, amnestic syndrome, residual and late onset psychotic disorder, other/ unspecified mental/ behavioural disorder) (94.3% primary or main diagnosis, 82.4% secondary diagnosis). As opioid consumption is almost always accompanied by dependence (F11.2), the following analyses focus on this diagnostic group (F11.2).

**Graph 1:**
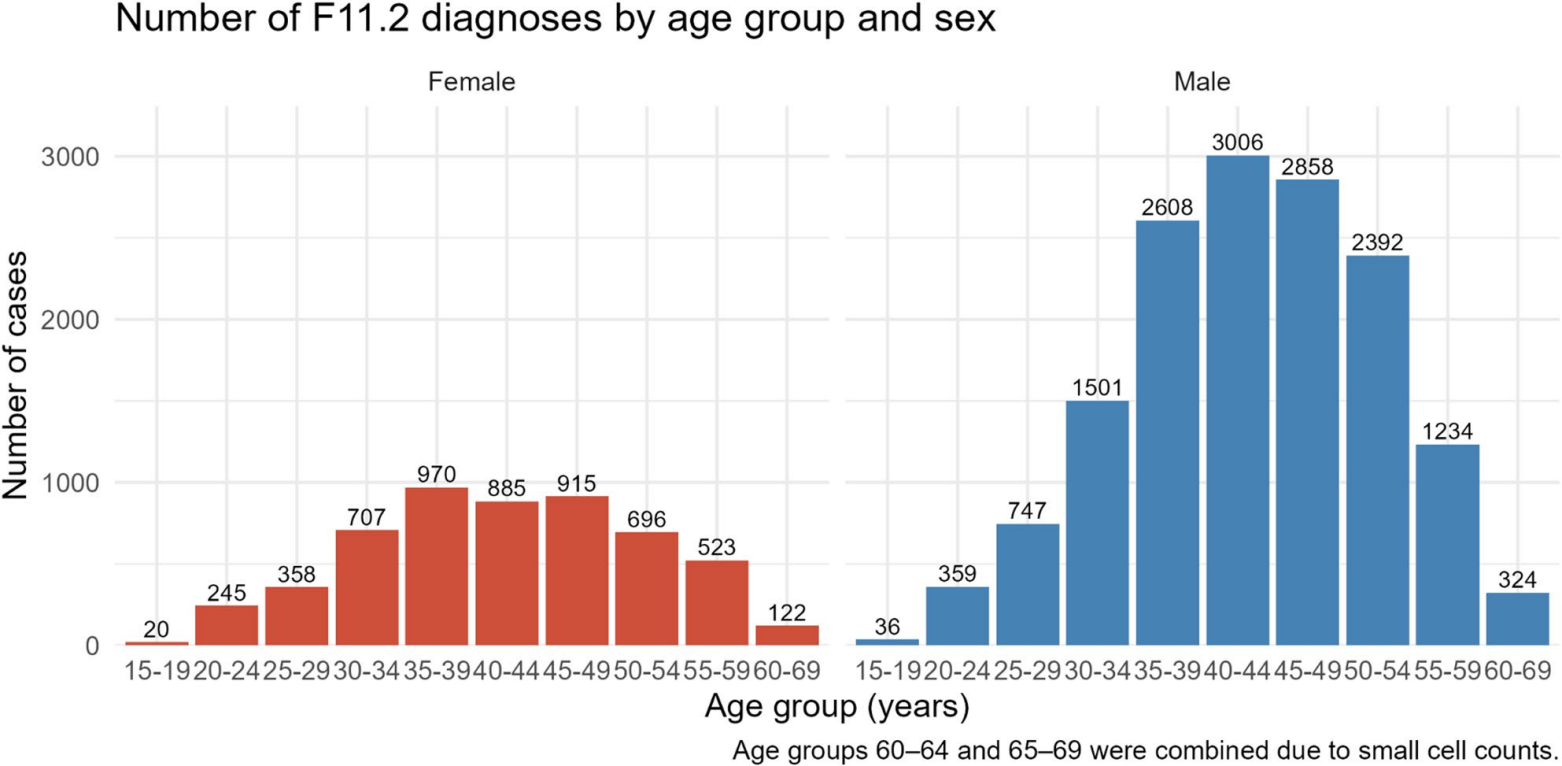
Number of diagnoses by age group and sex

Mean age of opioid dependent clients was 40.7 years (+/- 9.4 years). Male clients had a mean age of 41.1 years (+/- 9.1 years) whereas female clients had a mean age of 39.8 years (+/- 10.0 years). For more detailed information about sex and age distribution, please see graph 1.

### Specifics of age and sex

Among the 14,498 individuals of both sexes and within the complete age span suffering from a F11.2 main diagnosis, 10,722 (74.0%) were male and 3,776 (26.0%) were female. For secondary diagnoses (*N* = 6,045), 4,375 (72.4%) diagnoses occurred in men and 1,670 (27.6%) in women. Considering all F11.2 diagnoses (main or secondary), 15,065 (73.5%) diagnoses concerned men and 5,441 (26.5%) concerned women. The age distribution differed slightly between sexes. Among women, the highest number of F11.2 diagnoses was observed in the 35–39-year age group (*n* = 970), followed by the 45–49-year age group. Among men, the highest number was observed in the 40–44-year age group.

### Longitudinal data

The number of F11.2 diagnoses by year was as follows: 2016: *n* = 2,327; 2017: *n* = 2,831; 2018: *n* = 3,401; 2019: *n* = 3,947; 2020: *n* = 3,978; 2021: *n* = 4,022. Poisson regression showed a statistically significant annual increase of 11.1% in the number of F11.2 diagnoses (β = 0.105; IRR = 1.11, 95% CI 1.10–1.12; *p* < 0.001).

### Comorbidities

Among individuals with a primary diagnosis of F11.2, the most frequent comorbid diagnoses were alcohol dependence (F10.2) (13.5%), dependence on sedatives/ hypnotics (F13.2) (8.2%), cannabinoid dependence (F12.2) (7.2%), other poly substance use (F19.2) (5.9%), cocaine dependence (F14.2) (5.6%) and depression (F32.9) (5.4%). The only frequent somatic comorbidity was chronic viral hepatitis (B18.2) (7.8%). In sum, 40.3% of the clients were in opioid maintenance treatment.

For those with a secondary F11.2 diagnosis, main primary diagnoses were alcohol dependence (F10.2) (28.8%), other or polysubstance use (F19.2) (17.6%), paranoid schizophrenia (F20.0) (8.8%), depressive episode (F32.9) (8.3%) and recurrent depressive episode (F33.1) (5.9%).

For additional details, please see Table 2 (Table 2a/ b).

**Table 2:** Top 10 co-morbidities

**Secondary diagnosis**	***N*** ** (%)**
**a: Top 10 secondary diagnoses when F11.2 is main diagnosis**	
Other specified medical care (Z51.8)	3,554 (40.3%)
Alcohol dependence (F10.2)	1,192 (13.5%)
Sedative/hypnotic dependence (F13.2)	724 (8.2%)
Chronic viral hepatitis C (B18.2)	690 (7.8%)
Cannabinoid dependence (F12.2)	633 (7.2%)
Multiple/other psychoactive substance dependence (F19.2)	521 (5.9%)
Cocaine dependence (F14.2)	497 (5.6%)
Depressive episode, unspecified (F32.9)	475 (5.4%)
Harmful alcohol used (F10.1)	270 (3.1%)
Harmful cannabinoids use (F12.1)	254 (2.9%)
**Main diagnosis**	**N (%)**
**b: Top 10 main diagnoses when F11.2 is secondary diagnosis**	
Alcohol dependence (F10.2)	972 (28.8%)
Multiple/other psychoactive substance dependence (F19.2)	593 (17.6%)
Other specified medical care (Z51.8)	406 (12.0%)
Paranoid schizophrenia (F20.0)	296 (8.8%)
Depressive episode, unspecified (F32.9)	281 (8.3%)
Persistent somatoform pain disorder (F45.4)	206 (6.1%)
Recurrent depressive disorder, current episode moderate (F33.1)	198 (5.9%)
Moderate depressive episode (F32.1)	150 (4.4%)
Emotionally unstable personality disorder (F60.3)	138 (4.1%)
Recurrent depressive disorder, current episode severe without psychotic symptoms (F33.2)	131 (3.9%)

The diagnosis “Other specified medical care (Z51.8)” refers to opioid maintenance treatment

### Ability to work

Regarding daily work capacity, 58.3% (*n* = 11,962) of individuals were able to work less than 3 h per day, 10.0% (*n* = 2,051) were able to work 3–6 h per day, and 17.4% (*n* = 3,568) were able to work more than 6 h per day. For 14.3% (*n* = 2,925) of individuals, information on work capacity was not specified.

Among male clients, 57.3% (*n* = 8,637) were able to work less than 3 h per day, 9.8% (*n* = 1,477) were able to work 3–6 h per day, and 18.3% (*n* = 2,757) were able to work more than 6 h per day; for 14.6% (*n* = 2,194), work capacity was not specified.

Among female clients, 61.1% (*n* = 3,325) were able to work less than 3 h per day, 10.5% (*n* = 574) were able to work 3–6 h per day, and 14.9% (*n* = 811) were able to work more than 6 h per day; for 13.4% (*n* = 731), work capacity was not specified.

**Graph 2:**
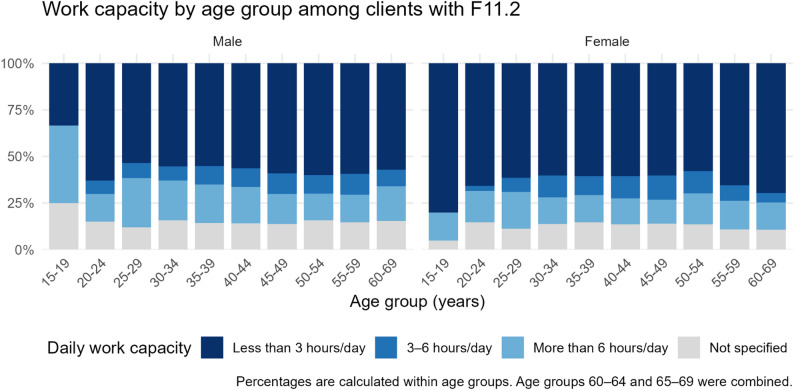
Work capacity by age group and sex

Regarding work capacity, sex differences as well as differences between age groups were observed. For a comprehensive overview, please see graph 2.

## Discussion

Job loss and unemployment are among the most stressful life events with detrimental effects on mental health [34], and, conversely, mental health disorders significantly increase the risk of becoming unemployed [31]. Previous analyses from this group [35, 36, 39, 46] demonstrated the significant association of unemployment with psychiatric disorders, especially mood disorders, neurotic, stress-related and somatoform disorders and substance use disorders, especially alcoholism.

Previous studies have shown that substance use disorders are frequent in German unemployed people (22%) [41]. While the relevance of alcohol use disorders for poor work and health performance is firmly established, the literature on opioid use disorder and employment status is rather limited [10, 41, 42, 46]. A special issue of “New Solutions”, a journal dedicated to environmental and occupational health policy, states in 2021 that “The workplace has been a neglected element” regarding OUD [47]. This must always be considered when searching for suitable literature to discuss the findings of this paper. This means that the present study has a pilot character and provides initial scientific evidence regarding the association of OUD, OUD treatment and labour-market aspects such as labour-market integration. Reduction of unemployment in OUD is not only an economic issue but also a significant psychosocial issue. Multiple lines of evidence show that both unemployment and engagement in illegal income-generating activities are associated with poorer outcomes in OUD [7] while decreased unemployment at least in HIV-positive drug users predicts decreased mortality [12], among others.

In this study, 22,683 assessments (0,5%) of socio-medically assessed FEA clients (total of *N* = 4,249,028) with at least one opioid-related ICD-10 F.11.x diagnosis between 2016 and 2021 were identified and included for further analysis. With respect to primary and secondary diagnosis, an additional mental disorder as first diagnosis was coded in 15,382 and in 7,340 clients as second diagnosis. Among the ICD-10 F11 diagnoses, opioid dependence (F11.2) was by far the most frequent subtype. Therefore, the subsequent analyses focused on this diagnostic group. Opioid dependence (F11.2) accounted for 94% of primary and 82% of secondary OUD diagnoses. The sex ratio male/ female of 3:1 reflects the prevalence of opioid use disorder in both sexes in Germany [48]. Interestingly, the dataset indicates an increasing number of F11.2 diagnoses by year from 2016 (*n* = 2,327) to 2021 (*N* = 4,022), while the number of socio-medical assessments by the MS of the FEA remained relatively constant during these years. Thus, the figures nearly doubled in 5 years. Whether this is due to changing consumption patterns or changes in the social environment remains unclear, as data are still lacking. To date there is no evidence for increasing opioid use in Germany over the last decade [48].

With respect to daily work capacity and functioning, 58.3% of individuals were found only to be able to work less than 3 h per day, 10.0% were able to work 3–6 h per day, and 17.4% were able to work more than 6 h per day. Even if there is a paucity of systematic literature, there is some evidence suggesting that medical professionals dealing with OUD patients do not pay enough attention to work capacity and labour affairs of their patients [47, 49].

Overall, differences between men and women were small. The largest differences were observed in individuals younger than 20 years; however, this age group was small and therefore did not allow meaningful interpretation. Work capacity was highest among individuals aged 25–29 years, possibly reflecting less advanced disease and greater treatment adherence. However, these explanations remain speculative because of the limited literature available on this topic. In sum, most of the unemployed individuals with opioid use disorders have no or very limited work capacity, far lower than observed for other mental disorders (about 40% with full ability to work [39].

Interestingly, somatic disorders, otherwise very prevalent in opioid use disorders [1, 6], were not frequently recorded as accompanying primary or secondary diagnoses, with the exception of hepatitis. Instead, the database stresses the role of other substance use disorders such as alcohol use disorders, and other mental disorders (depression, schizophrenia). In other words: substance (opioid) use per se was the dominant reason for unemployment, not specific medical disorders associated with substance use. Multiple lines of evidence suggest that comorbidity in patients with OUD is high [8]. National reports in treatment seeking individuals indicate that 37.9% of them also meet diagnostic criteria for a comorbid psychiatric disorder [17].

Little is known on the costs and economic burden of OUD in Germany. There are some data on the direct costs for health treatment. Reimer et al. [50] estimated the annual sickness funds costs on average at 7,470 Euro for treatment with opioid agonists. Neusser et al. [51] examined expenditures of the statutory health insurance as well as the German pension funds for the base year 2016 and estimated the total expenditure amounted to approximately 685,274,000 Euro. A recent extensive Norwegian study [52] found that OAT, the undisputed gold standard in treatment of OUD [25] is cost effective.

This study has some important limitations. First, this is a purely statistical analysis and only unemployed individuals who underwent socio-medical assessment by the FEA were included. The study does not allow to draw any conclusions about the mental health status of the entire population of unemployed people in Germany (see [39]). Second, the database only provides information about the two main diagnoses; further psychiatric/ somatic diagnoses are not available. It can therefore be assumed that the prevalence rates of comorbid disorders were even higher. Third, this is a cross-sectional study and only data from 2016 to 2021 was available for analysis due to technical registration requirements. Longitudinal assessments over a longer time period are necessary to further elucidate employment status and ability to work over time. Fourth, no individual psychometric or clinical data beyond those given were available for analysis. The assessment itself follows a defined protocol and is identical for all individuals assessed; however, some clients were assessed based upon medical data files solely while others underwent additional (physical) socio-medical assessment. Further sociodemographic, health-related and occupational data about the unemployed persons could not be included due to German data protection regulations. Thus it is not possible to draw firm conclusions about the progress, interactions and outcomes of unemployment and opioid use disorders. Fifth, the potential impact of the COVID pandemic, which began in 2020, should be considered, as it may have affected employment status, although it is unlikely to have influenced work functioning.

Regarding specific needs of and recommendations for the target group and the labour market (re-) integration process, it has to be stated that OUD is not a very frequent but a severe and significant health aspect that has to be considered regarding the whole reintegration process and beyond. It could be advisable to establish a closer relationship between labour market and health policy to establish tailored interventions to these specific clients and their specific needs during the (re-) integration process and to support them as employees and patients as well as to support their employers. Since OUD can have serious long-term consequences (e.g. reduced working capacity) and require lifelong treatment, the time after the (re-) integration process has to be considered as well; in this respect, part-time work, supported employment and other (rehabilitation) models could be used. In sum, OUD therapy should not be considered limited to medical treatment but rather should be understood within a broader psychosocial and labour-market context.

## Conclusions

In conclusion, this study provides basic data on frequency of opioid use disorders in unemployed people in Germany and suggests comparatively low ability to work. The role of other substance use and mental disorders is apparent. The number of studies focusing on employment rate and especially work ability in opioid users is surprisingly limited [8, 10] and basically restricted to the role of opioid maintenance drugs [25]. Since unemployment and socioeconomic deprivation are risk factor for OUD [53], a better understanding of factors associated with unemployment and poor work capacity in patients with OUD may also provide better therapeutic options and outcomes in affected individuals.

## Data Availability

The data that support the findings of this study are available from the Medical Service of the Federal Employment Agency but restrictions apply to the availability of these data, which were used under license for the current study, and so are not publicly available. Data are however available from the authors upon reasonable request and with permission of the Data Protection Department of the Federal Employment Agency.
